# Supplementary material for: Absence of bulk charge density wave order in the normal state of UTe2
Source: Nat Commun. 2024 Nov 9;15:9713. doi: 10.1038/s41467-024-53739-8 (PMC11550464; doi:10.1038/s41467-024-53739-8)
Supplement: Supplementary file 1 — Supplementary Information [file 41467_2024_53739_MOESM1_ESM.pdf]

Supplementary Material for Absence of bulk charge density wave order in the normal state of UTe_2

C. S. Kengle[1,2] et. al.

The following Supplementary Material contains:

- Supplementary Text S1 - S6
- Supplementary Figures S1 - S3
- Supplementary Table S1

TEXT S1: FOURIER TRANSFORMATION OF STM IMAGES

STM images, reported by *Gu et al, LaFleur et al., Aishwaria et al.* [23, 24, 25], were taken on freshly cleaved (011) surfaces. The top layer exposed through cleaving was composed of Te-atoms and is illustrated schematically in Fig. S1(a) by a gray plane. Within this plane, Te-atoms form an orthorhombic lattice with centered rectangular unit cell (see (b)) of lattice parameters $\alpha_1 = a$ and $\alpha_2 = \sqrt{b^2 + c^2}$.

Fig. 1(a) of the main text illustrates schematically the fast Fourier transformations of typical STM images taken below the transition temperature. In this schematic illustration, wave-vectors are characterized in the basis $\mathbf{Q} = i \cdot \beta_1 + j \cdot \beta_2$, where $\beta_1 = (2\pi/\alpha_1, 0)$ and $\beta_2 = (0, 2\pi/\alpha_1)$. Fig. S1(c) shows the indexation of several structural peaks at low Q in this basis.

TEXT S2: CALCULATION OF CHARGE-DENSITY WAVE-VECTORS IN BULK

We required on the one hand that the three wave-vectors $\mathbf{q}_1$, $\mathbf{q}_2$, and $\mathbf{q}_3$ possess the projections q_1, q_2, and q_3. The sets of reciprocal-space points with projections q_1, q_2, and q_3, respectively, correspond to lines l_1, l_2, and l_3 that are parallel to the direction (011). On the other hand, we assumed that the bulk wave-vectors are located on the same reciprocal-space surface as the points W, L_1, and L_2, which are characterized by a modulation period of two crystallographic units along (011), i.e., $d_{(011)}$. $\mathbf{q}_1$, $\mathbf{q}_2$, and $\mathbf{q}_3$ therefore correspond to the points on the lines l_1, l_2, and l_3, where $\mathbf{q}_i \cdot (\mathbf{a}_2 + \mathbf{a}_3) = 2 \cdot \pi$.

TEXT S3: EXPERIMENTAL SETUP

The setup of diffraction experiments, carried out at the beamline EH2 of the station P09 (DESY, Hamburg), is presented in Fig. S2. The polarization, which at P09 initially comes lying in the scattering plane (denoted π-polarization), was rotated by ninety degrees using phase plates, such that incident X-rays probing the sample had linear polarization perpendicular to the scattering plane (denoted σ-polarization).

The polarization-dependent scattering amplitudes for Thomson charge-scattering in the two channels $\pi\pi'$ and $\sigma\sigma'$, respectively, are given by $f_{\sigma\sigma'} = F^{(0)}$ and $f_{\pi\pi'} = F^{(0)} \cdot \cos(2\theta)$, where $F^{(0)}$ corresponds to a material specific parameter and 2θ denotes the scattering angle, thereby guaranteeing maximum scattering amplitudes for $\sigma\sigma'$-scattering, whereas for $\pi\pi'$ charge scattering may be suppressed (cf. Ref. [36]).

Integrated intensities of resolution limited structural Bragg peaks, $\mathbf{G}$, as observed in our experiments, are essentially given by $I(\mathbf{G}) = A(\mathbf{G}) \cdot V(\mathbf{G})$, where $A(\mathbf{G})$ (or $A_{\mathbf{G}}$) denotes the amplitude and $V(\mathbf{G})$ (or $V_{\mathbf{G}}$) the volume of experimental momentum-space resolution (cf. Methods). Typical values observed were $V_{(0,1,1)}^{3.37\mathrm{keV}} = 8.67 \times 10^{-9} \mathrm{\AA}^{-3}$ and $A_{(0,1,1)}^{3.37\mathrm{keV}} = 4 \cdot 10^4$ cts/s for $(0,1,1)$ Bragg peak measured at 3.37 keV, or $V_{(022)}^{4.94\mathrm{keV}} = 3.47 \times 10^{-9} \mathrm{\AA}^{-3}$ and $A_{(022)}^{4.94\mathrm{keV}} = 7 \cdot 10^8$ cts/s for $(0,2,2)$ peak measured at 4.94 keV. Note that throughout the text, X-ray intensities are given as cts/s after correcting for attenuation factors.

TEXT S4: RESOLUTION AND ACCURACY OF THE DIFFRACTOMETER

The experimental $\mathbf{Q}$-resolution in our REXS experiments on P09 may be inferred from the width of resolution-limited structural Bragg peaks. At 3.73 keV incident X-ray energy, around the M_4 edge of uranium, the profile of $\mathbf{Q} = (0, 1, 1)$ is Gaussian with FWHMs along the axes $(1, 0, 0)$, $(0, 1, 1)$, and $(0, -0.39, 2.03)$ given by $7.14 \cdot 10^{-4}\,\text{Å}^{-1}$, (corresponding to 0.000473 r.l.u.), $2.64 \cdot 10^{-3}\,\text{Å}^{-1}$, and $4.6 \cdot 10^{-3}\,\text{Å}^{-1}$, respectively. At 4.924 keV incident energy, around the L_1 edge of tellurium, the FWHMs of $\mathbf{Q} = (0, 2, 2)$ along the axes $(1, 0, 0)$, $(0, 1, 1)$, and $(0, -0.39, 2.03)$ are given by $6.2 \cdot 10^{-4}\,\text{Å}^{-1}$, $1.1 \cdot 10^{-3}\,\text{Å}^{-1}$, $5.1 \cdot 10^{-3}\,\text{Å}^{-1}$, respectively.

Systematic errors in hkl-positions due to inaccuracies in angular precision of the diffractometer were relatively low. The resolution is larger for smaller incident X-ray wavelengths and larger momentum transfers. We find that at the tellurium L_1 edge (4.94 keV), maximum deviations in momentum transfers observed were $\delta_{\mathbf{Q}} = 0.0056\,\text{Å}^{-1}$ at the $\mathbf{Q} = (2, 3, 3)$ structural Bragg peak. At the uranium M_4 edge (3.73 keV), the maximum inaccuracies amounted to $\delta_{\mathbf{Q}} = 0.00058\,\text{Å}^{-1}$, as observed at the $\mathbf{Q} = (0, 1, 1)$ Bragg peak.

TEXT S5: LAUE DIFFRACTION IMAGE OF SAMPLE

Fig. S3 shows a Laue image taken on the (011) surface of one of our samples.

TEXT S6: CRYSTAL STRUCTURE FACTOR

For the calculation of the crystal structure factor of UTe_2, we consider a primitive set of atoms given in Table S1. The Fourier transformed scattering length for a unit cell is given by:

$$F(\mathbf{Q}) = \sum_{a\sim \mathrm{U}} f_{\mathrm{U}}(\mathbf{Q}) \exp(\mathrm{i}\cdot\mathbf{Q}\cdot\mathbf{R}) + \sum_{a\sim \mathrm{Te1}} f_{\mathrm{Te}}(\mathbf{Q}) \exp(\mathrm{i}\cdot\mathbf{Q}\cdot\mathbf{R}) + \sum_{a\sim \mathrm{Te2}} f_{\mathrm{Te}}(\mathbf{Q}) \exp(\mathrm{i}\cdot\mathbf{Q}\cdot\mathbf{R})\,, \tag{1}$$

where the sum for each atomic type goes over the atomic positions given in Tab. S1. The $\mathbf{Q}$-dependent scattering lengths of U and Te, f_{Te} and f_{Te}, were calculated in Gaussian approximation with the parameters listed in the International Tables for Crystallography. As there were no tabulated values for Te-ions, we approximated the crystal as a composition of neutral atoms.

The structure factor was obtained using the relation $S(\mathbf{Q}) = F^{*}(\mathbf{Q}) \cdot F(\mathbf{Q})$.

FIGURE S1

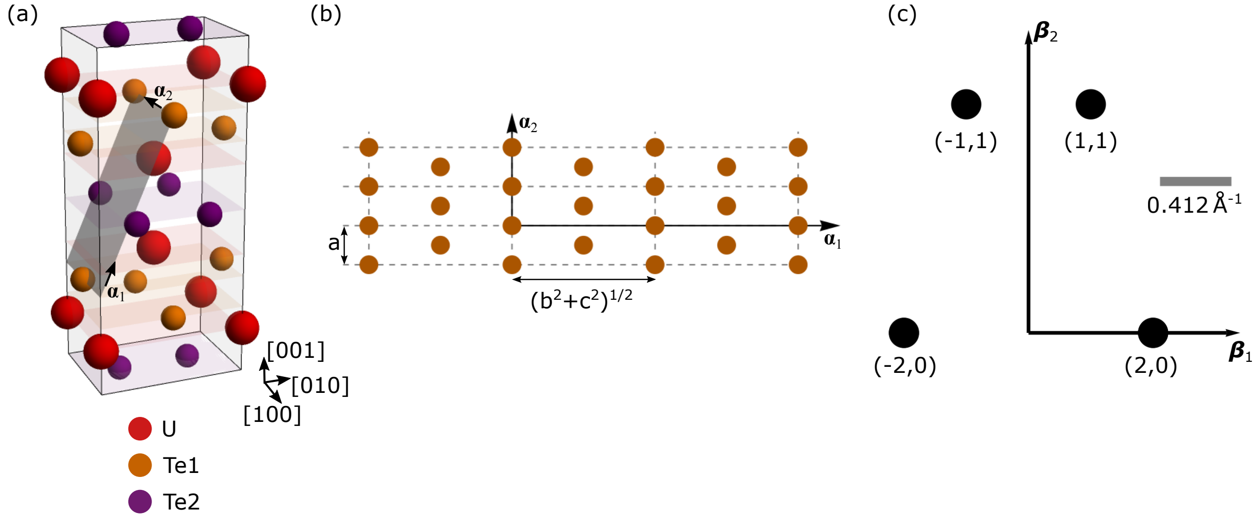

FIG. S1. **Two-dimensional Fourier transformation describing the periodicity of Tellurium atoms in (011) crystallographic planes.** (a) Conventional crystallographic unit cell of UTe_2. Uranium atoms (Wyckoff position 4i) are shown as red spheres. Tellurium atoms at Wyckoff positions 4h (Te2) and 4j (Te1) are shown as purple and orange spheres, respectively. The gray rectangle indicates a $(01\bar{1})$ plane passing through Te1 atoms. Te2-atoms are slightly shifted out of the plane. (b) In the Te-plane illustrated in (a), Te1-atoms form a centered rectangular lattice. Te2-atoms are arranged on the same lattice, but are not in the same plane as the Te1-atoms. (c) Schematic view of Fourier transformed STM images on a (011) Te-plane. Shown in terms of black circles is the indexation of several Fourier components at low momentums, reflecting the periodicity of Te1-atoms (and of Te2-atoms).

FIGURE S2

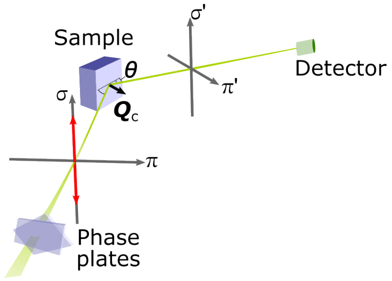

FIG. S2. **Schematic illustration of the experimental setup.** The schematic figure illustrates the X-ray diffraction setup at P09. Diffraction experiments were carried out in reflection geometry. The situation in the figure is for putative Bragg diffraction with momentum transfer $\mathbf{Q}_c$. The respective scattering angle is given by 2θ. The path of X-rays is illustrated in green shading. Incident X-rays after the phase plates had linear σ polarization (red arrow). For X-rays after the scattering process, the polarization components are primed. Accordingly, polarization of scattered X-rays in and perpendicular to the scattering plane are denoted π' and σ', respectively.

FIGURE S3

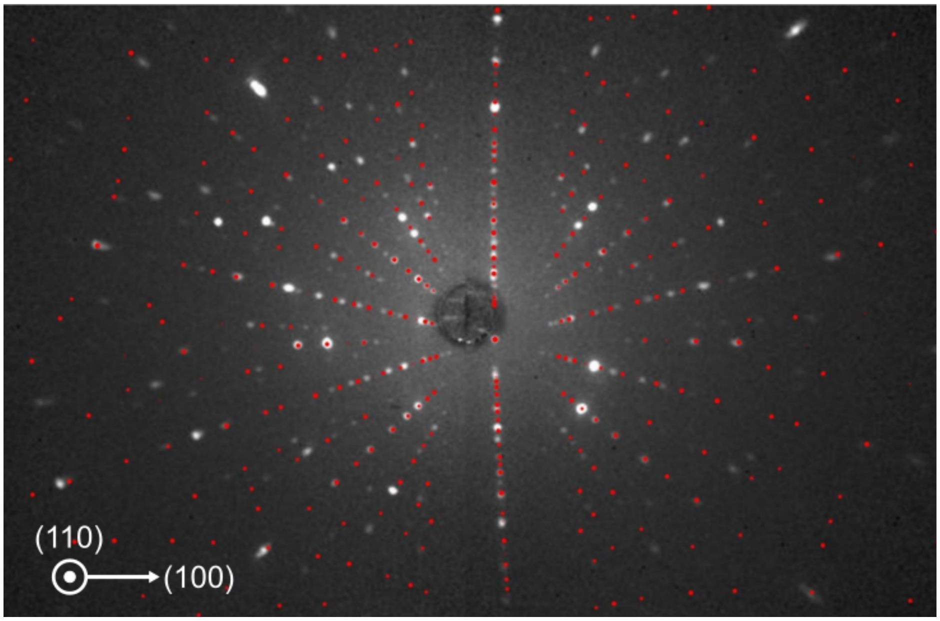

FIG. S3. **Laue Diffraction Pattern of UTe_2 sample studied.** The Laue picture was taken on a surface (011). The sample was aligned such that the (100) direction was horizontal. The red points indicate the indexation of the Laue pattern.

TABLE S1

Atom label	WP	Coordinates
U	4i	(0,0,0.13523) , (0,0,0.86477), (0.5,0.5,0.36477), (0.5,0.5,0.63523)
Te1	4j	(0.5,0,0.29779),(0.5,0,0.70221),(0,0.5,0.79779),(0,0.5,0.20221)
Te2	4h	(0,0.2508,0.5),(0,0.7492,0.5),(0.5,0.7508,0),(0.5,0.2492,0)

TABLE S1. Primitive set of atoms describing the crystal structure of UTe_2. The first column provides the atom labels (atom type + number), the second column the Wyckoff position, and the third column the coordinates of atoms in the conventional orthorhombic basis.
